# Characterization and anti-biofilm activity of bacteriophages against urinary tract *Enterococcus faecalis* isolates

**DOI:** 10.1038/s41598-022-17275-z

**Published:** 2022-07-29

**Authors:** Doaa M. El-Atrees, Reham F. El-Kased, Ahmad M. Abbas, Mahmoud A. Yassien

**Affiliations:** 1 Department of Microbiology, Faculty of Pharmacy, The British University in Egypt (BUE), El-Sherouk City, 11837 Cairo Egypt; 2 Department of Microbiology and Immunology, Faculty of Pharmacy, Ain Shams University, African Union Organization Street, Abbasia, Cairo, 11566 Egypt; 3 Department of Microbiology and Immunology, Faculty of Pharmacy, King Salman International University, Sinai, Egypt

**Keywords:** Drug discovery, Microbiology

## Abstract

Strong biofilm-forming *Enterococcus feacalis* urinary tract pathogens (n = 35) were used to determine the lytic spectrum of six bacteriophages isolated from sewage samples. Only 17 *Enterococcus feacalis* isolates gave lytic zones with the tested bacteriophages from which five isolates were susceptible to all of them. The isolated enterococcal phages are characterized by wide range of thermal (30–90 °C) and pH (3–10) stability. They belong to order *Caudovirales,* from which four bacteriophages (EPA, EPB, EPD, EPF) belong to family M*yoviridae* and two (EPC, EPE) belong to family *Siphoviridae.* In addition, they have promising antibiofilm activity against the tested strong-forming biofilm *E. faecalis* isolates. The enterococcal phages reduced the formed and preformed biofilms to a range of 38.02–45.7% and 71.0–80.0%, respectively, as compared to the control. The same promising activities were obtained on studying the anti-adherent effect of the tested bacteriophages on the adherence of bacterial cells to the surface of urinary catheter segments. They reduced the number of adherent cells to a range of 30.8–43.8% and eradicated the pre-adherent cells to a range of 48.2–71.1%, as compared to the control. Overall, the obtained promising antibiofilm activity makes these phages good candidates for application in preventing and treating biofilm associated *Enterococcus faecalis* infections.

## Introduction

*Enterococcus* species are commensals gram-positive bacteria which cause many infections in susceptible hosts, such as endocarditis, bacteremia, oral infections and urinary tract infections (UTI) especially device associated infections. In Egyptian UTI patients, *Enterococcus faecalis* (*E. faecalis*) is found to be the second highest prevalence after *Escherichia coli*. UTIs in hospitalized, catheterized and diabetic patients are higher than outpatients^1,2^. One of the virulence factors of *Enterococcus* besides the enzymes and proteins secreted is its ability to form biofilm^3^.

The National Institutes of Health (NIH) revealed that among all microbial and chronic infections, 65% and 80%, respectively, are associated with biofilm formation. These include both, device and non-device-associated infections^4^. Catheters associated urinary tract infections (CAUTI) account for 40% of all nosocomial infections, of which 15% to 30% are associated with *E. faecalis* and *Enterococcus faecium *(*E. faecium*)^5,6^.

*E. faecalis* lives as commensal organism in the gastrointestinal tract. Nonetheless, it may trigger life-threatening infections including endocarditis, bacteremia, UTI and meningitis, and it is particularly common in hospitals where antibiotic resistance has grown^7^. The devices associated enterococcal infections*,* like CAUTI, are mainly due to biofilm associated bacteria^8^.

Biofilm forming bacteria show resistance to many antibiotics and immune response which results in treatment failure^9^. Given the difficulty of treating and eradicating biofilm associated infections, there is an unmet need for therapeutic options other than antibiotics to prevent biofilm formation. New approaches were developed to overcome this problem, among which was bacteriophage therapy^10^.

Bacteriophages are the natural predators for bacteria, and are the most common biological entities on the planet; phage particles are usually five to ten times more abundant than bacterial cells. So, phage predation is thought to cut the global bacterial population in half every 48 h^11^. Bacteriophages have many advantages over antibiotics therapy; they can affect all types of bacteria including multi drug resistant ones, they are promising in the economic aspect, and finally, being natural products, they are likely to be devoid of apparent toxicity^12^. These characteristics make bacteriophages an attractive non-antibiotic therapy for treating biofilm associated bacterial infections^13^.

The aim of the present study was to investigate the effect of different isolated bacteriophages on the planktonic and biofilm associated *E. faecalis* urinary tract pathogens. To achieve this, bacteriophages were isolated from sewage, followed by their characterization, and in vitro assessment of their lytic and antibiofilm activity against biofilm associated *E. faecalis* urinary tract pathogens.

## Results

### Identification of bacterial isolates and assessment of biofilm formation

All the collected clinical isolates (n = 100) can grow on Bile Esculin agar. In addition, the isolates are gram positive and catalase positive confirming that all the isolates are *Enterococcus* species.

The results of evaluating the biofilm formation showed that 35 isolates formed strong biofilm (OD range 0.24–2.66) (Supplementary Table S1), while the other isolates were either moderate (n = 29) or weak/non-biofilm forming (n = 36). The identification of the strong biofilm producer isolates (n = 35) was further confirmed by VITEK and the results showed that all the isolates were *Enterococcus faecalis*.

### Isolation and purification of bacteriophages from sewage samples

Six phages were isolated from the community samples, of which four bacteriophages (EPA, EPB, EPC, EPD) were isolated from Kalyoub governorate’s samples and two (EPE, EPF) from Zagazig governorate’s samples. On the other hand, no bacteriophage could be isolated from hospitals and clinics’ samples. The isolated bacteriophages were identified by formation of clear plaques on bacterial lawns. Four phages showed hallow zone around the plaques and the other two showed plaques with defined boundaries.

### Characterization of bacteriophages

#### Determination of host range

When the isolated phages were spotted on the surface of plates seeded with the strong biofilm forming isolates (n = 35), only 17 bacterial isolates gave clear plaques from which five bacterial isolates were susceptible to all the tested bacteriophages (Table 1).

**Table 1:** Lytic activity spectra of 6 different enterococcal phages (EPA: F) against different enterococcal isolates (n = 17).

Isolates codes	EPA	EPB	EPC	EPD	EPE	EPF
EF11	–	–	√	√	√	√
EF16	√	√	√	√	–	–
EF20	–	–	–	√	–	–
EF38	√	√	√	√	√	√
EF43	√	√	√	√	√	√
EF48	√	√	–	–	–	–
EF80	–	–	√	–	√	–
EF99	–	–	√	√	√	–
EF101	√	√	√	√	√	√
EF103	√	√	–	√	√	–
EF113	√	–	√	√	√	–
EF114	–	√	√	√	√	√
EF125	√	√	–	√	√	√
EF133	√	√	√	√	√	√
EF134	√	√	√	√	√	√
EF146	√	–	–	–	–	–
EF151	√	–	√	√	–	–

Isolate codes (EF)*: are the codes for *E. faecalis* isolates.

(√) indicates bacterial lysis, (–) indicates absence of plaques (no lysis).

#### Plaque morphology

**Figure 1:**
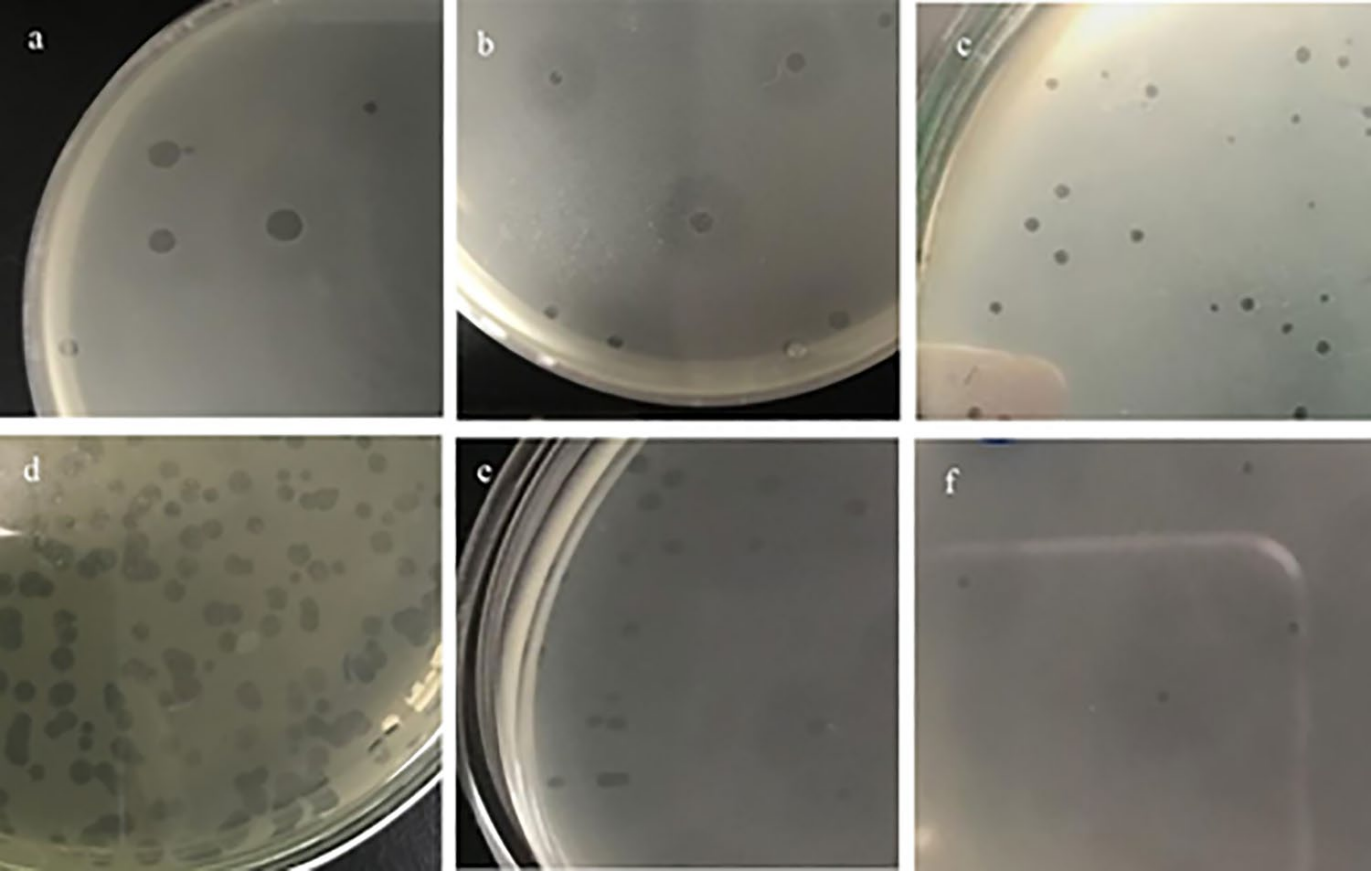
Plaques pictures of the 6 bacteriophages (EPA: F), as formed on *Enterococcus* double layer agar plates. (**a**) EPA: large plaques (diameter: up to 4 mm) with hallow zones. (**b**) EPB: medium plaques (diameter: 2–3 mm) with hallow zone. (**c**) EPC: medium plaques (diameter: 1–2 mm) without hallow zones. (**d**) EPD: large plaques (diameter: up to 4 mm) without hallow zone. (**e**) EPE: medium plaques (diameter: 1 mm) with hallow zone. (**f**) EPF: pinpoint plaques (diameter: < 1 mm) with hallow zone.

The formed plaques are circular. Four bacteriophages (EPA, EPE, EPC, EPD) showed plaques with diameters of range 1–4 mm. while, the other two bacteriophages (EPB, EPF) produced plaques with diameters less than 1 mm (Fig. 1). Hallow zones around the plaques were produced by only four bacteriophages (EPA, EPB, EPE, EPF).

### Effect of temperature on bacteriophages’ stability

All the bacteriophages showed lytic activity against the five enterococcal isolates (EF38, EF43, EF101, EF133 and EF134), are sensitive to all the tested bacteriophages, after exposure to temperature 40, 50, and 70 °C for 1 h. On exposure to 90 °C, the three phages EPD, EPE and EPF caused promising lytic activity to all the tested isolates. In addition, only EPD phage induced lytic activity after exposure to 100 °C (Supplementary Table S2).

### Effect of pH on bacteriophages’ stability

The experiment was carried against the five enterococcal isolates that are sensitive to all the tested bacteriophages; EF 38, EF 43, EF 101, EF 133 and EF 134. The obtained results revealed that the six bacteriophages did not lose their infectivity after 1 h exposure to all the tested pH values (3–11).

### Transmission electron microscope visualization

**Figure 2:**
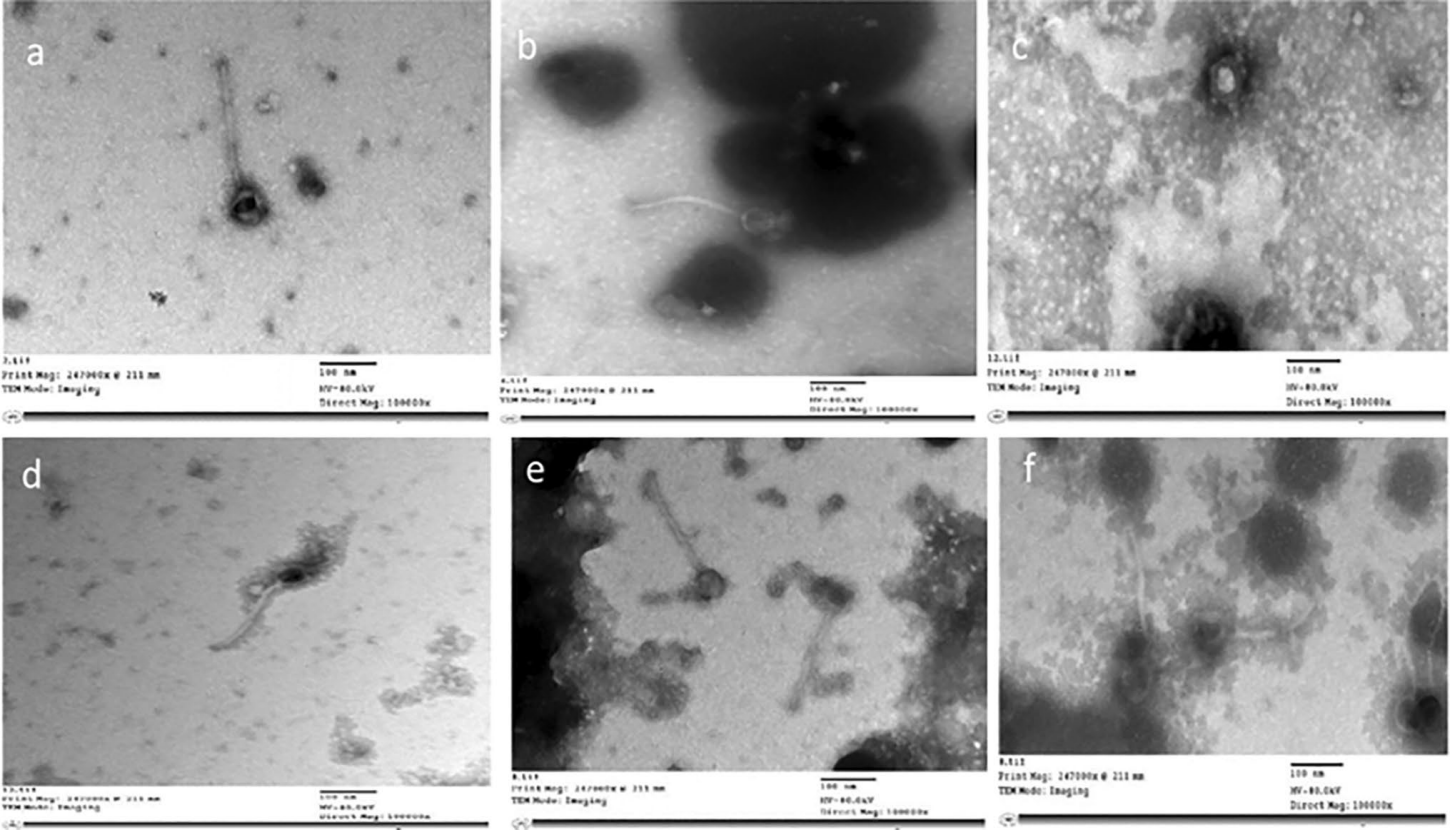
TEM images of the six different enterococcal bacteriophages (**a**) phage EPA, (**b**) phage EPB, (**c**) phage EPC, (**d**) phage EPD, (**e**) phage EPE, (**f**) phage EPF, with scale bar 100 nm.

According to the data of transmission electron microscope and the international committee on taxonomy of viruses, the 6 bacteriophages are tailed phages belong to order *Caudovirales,* from which 4 bacteriophages (EPA, EPB, EPD, EPF) belong to family M*yoviridae* and had heads (dimension ranges: 53.8–119 × 59.5–108 nm) attach to long and contractile tails (159–197 nm length and 9.03–13.6 nm width). In addition, the other two bacteriophages (EPC, EPE) belong to family *Siphoviridae* possess heads (dimension ranges: 48.6–63.8 × 53.1–62.8 nm) to which long, flexible and non-contractile tails (204–212 nm length and 5.97–9.9 nm width) were attached^14,15^ (Table 2, Fig. 2A–F).

**Table 2:** Dimensions of the six enterococcal phages (EPA: F) by using transmission electron microscope.

Bacteriophage	Head (nm)	Tail length × width (nm)
EPA	119 × 108	159 × 9.03
EPB	63.8 × 65.9	194 × 13.6
EPC	63.8 × 62.8	212 × 9.9
EPD	55.3 × 61.4	197 × 10.4
EPE	48.6 × 53.1	204 × 5.97
EPF	53.8 × 59.5	189 × 9.73

### Antibiofilm effect of bacteriophages

#### Prevention of biofilm formation

**Figure 3:**
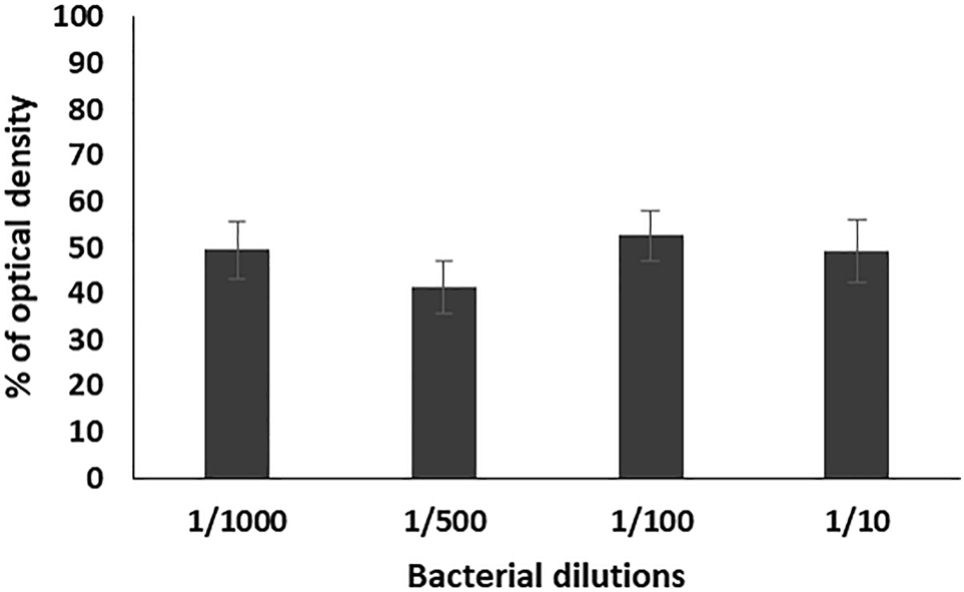
Percentage of optical density of the formed biofilms of 17 enterococcal isolates, prepared in different dilutions (1/10, 1/100, 1/500, and 1/1000) from bacterial suspension adjusted to 0.5 MacFarland, in the presence of the six bacteriophages (EPA, EPB, EPC, EPD, EPE, EPF) as compared to the control. Each bar represents the mean of three independent measurements.

Different bacterial dilutions (1/10, 1/100, 1/500, 1/1000) of the selected *Enterococcus* isolates (n = 17) were used against the six tested bacteriophages with titer 10^7^. Results were calculated as an average for every specific dilution and then as a collective average for the six bacteriophages. The results of antibiofilm of the tested bacteriophages showed that the average of percentages of the formed biofilms as compared to the control on using the prepared bacterial dilution were 49.3, 41.4, 52.6 and 49.5% respectively. The lowest percentage of biofilm formation was obtained by using 1/500 dilution (Fig. 3).

**Figure 4:**
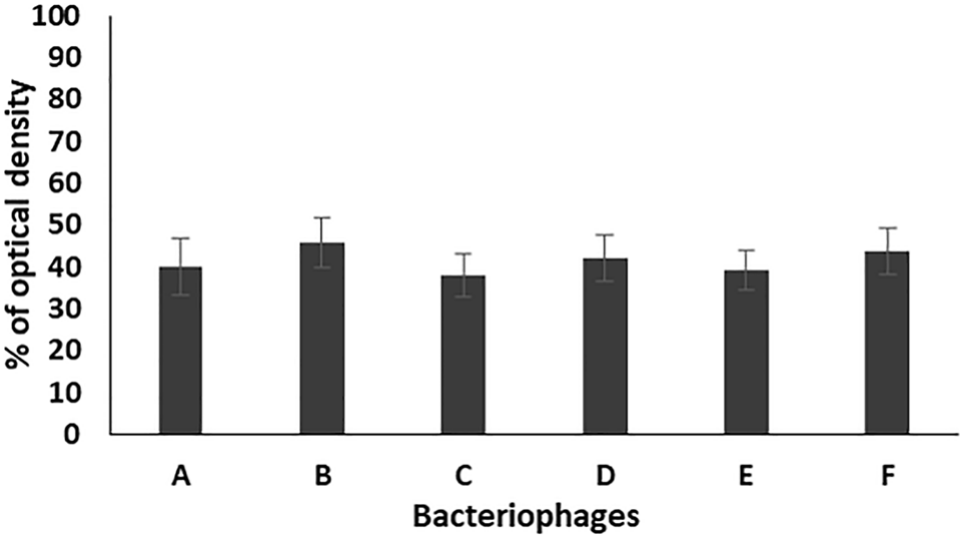
Percentage of optical density of the formed biofilms of 17 enterococcal isolates, prepared in 1/500 dilution from bacterial suspension adjusted to 0.5 MacFarland, in the presence of the six bacteriophages (A: EPA, B: EPB, C: EPC, D: EPD, E: EPE, F: EPF) as compared to the control. Each bar represents the mean of three independent measurements.

The results of antibiofilm effect of the tested bacteriophages (EPA, EPB, EPC, EPD, EPE, EPF) against the tested enterococcal isolates at dilution 1/500 showed reduction in the formed biofilms to 39.9, 45.7, 38, 42.1, 39.2, 44% as compared to that of the control, respectively (Fig. 4).

### Bacteriophage activity on preformed biofilm

**Figure 5:**
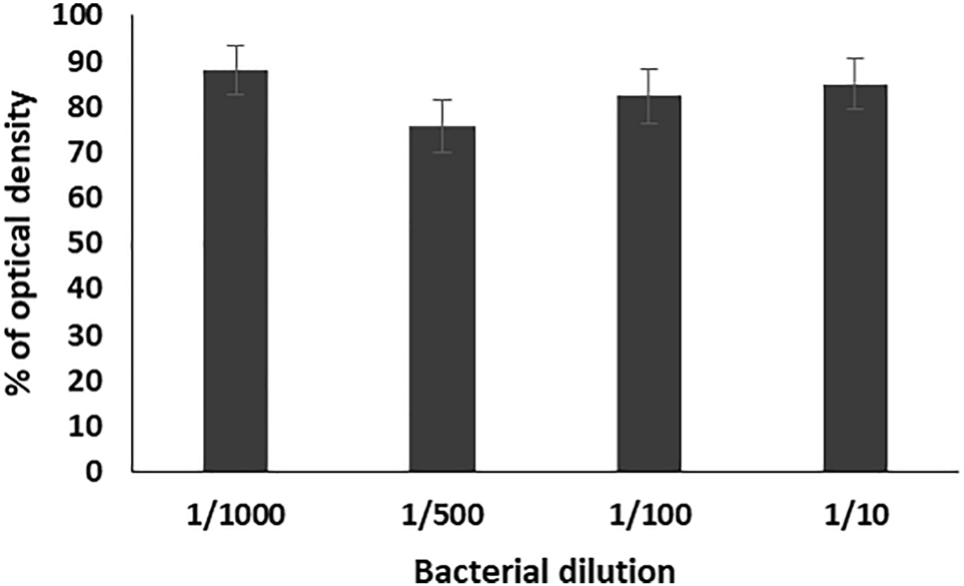
Percentage of the formed biofilms of 17 enterococcal isolates, prepared in different dilutions (1/10, 1/100, 1/500, and 1/1000) from bacterial suspension adjusted to 0.5 MacFarland, after treatment the preformed biofilms with the six bacteriophages (EPA, EPB, EPC, EPD, EPE, EPF) as compared to the control. Each bar represents the mean of three independent measurements.

Results were calculated as an average for every specific bacterial dilution and then as a collective average for the six bacteriophages. The obtained results showed that the tested bacteriophages caused reduction in the preformed biofilms by different bacterial dilutions 1/10, 1/100, 1/500, and 1/1000, to the averages of 85.1, 82.3, 76 and 88% as compared to the control, respectively. The lowest percentage of biofilm formation was obtained at dilution 1/500 (Fig. 5).

**Figure 6:**
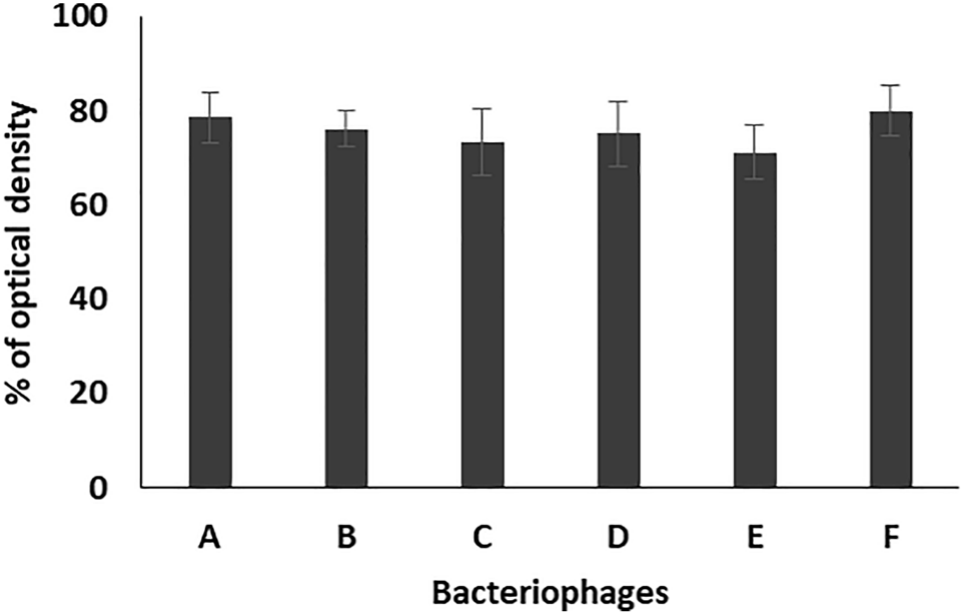
Percentage of the formed biofilms of 17 enterococcal isolates, prepared in different dilutions (1/10, 1/100, 1/500, and 1/1000) from bacterial suspension adjusted to 0.5 MacFarland, after treatment the preformed biofilms with the six bacteriophages (A: EPA, B: EPB, C: EPC, D: EPD, E: EPE, F:EPF) as compared to the control. Each bar represents the mean of three independent measurements.

This bacterial dilution (1/500) was used to evaluate the efficiency of the tested bacteriophages in eradicating the preformed biofilms by 17 enterococcal isolates. The obtained results revealed that the bacteriophages EPA, EPB, EPC, EPD, EPE, EPF caused reduction in the OD of the preformed biofilm to 78.4, 76, 73.3, 75, 71, 80% as compared to the control, respectively (Fig. 6).

### Effect of bacteriophage on bacterial adherence to urinary catheter surfaces

The three strongest biofilm producer enterococcal isolates (EF104, EF134, and EF151) and the phages (EPA, EPC and EPE) that showed the highest antibiofilm activity were used in this experiment.

**Figure 7:**
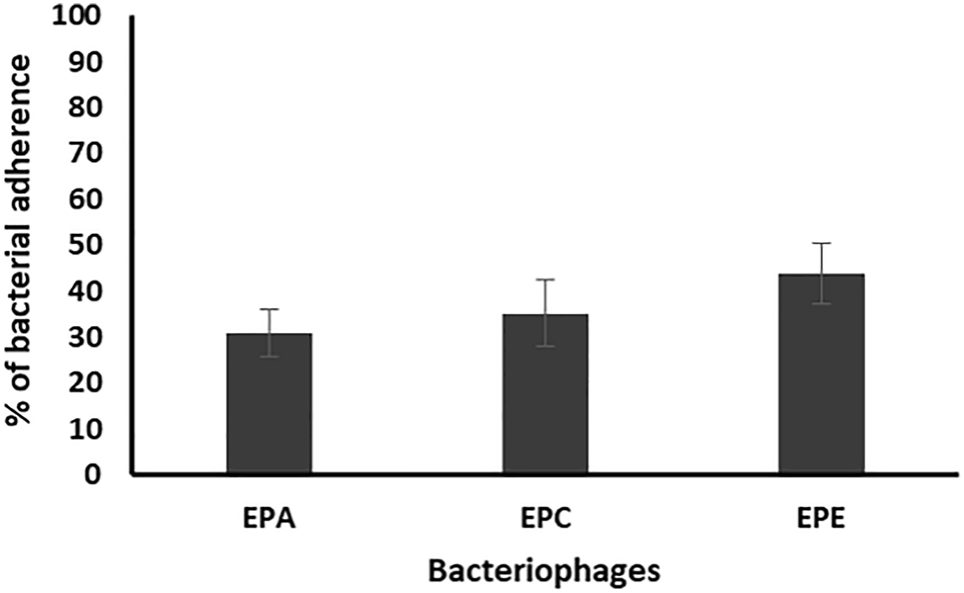
Percentage of bacterial adherence on the surface of catheters segments of 3 enterococcal isolates (EF104, EF134, and EF151), prepared in dilution 1/500 from bacterial suspension adjusted to 0.5 MacFarland, in the presence of the three bacteriophages (EPA, EPC, EPE) as compared to the control. Each bar represents the mean of three independent measurements.

The obtained results showed that the presence of the bacteriophages EPA, EPC and EPE reduced the number of adherent enterococcal cells to 30.8, and 35.3, 43.8%, respectively, as compared to that of the control (Fig. 7).

**Figure 8:**
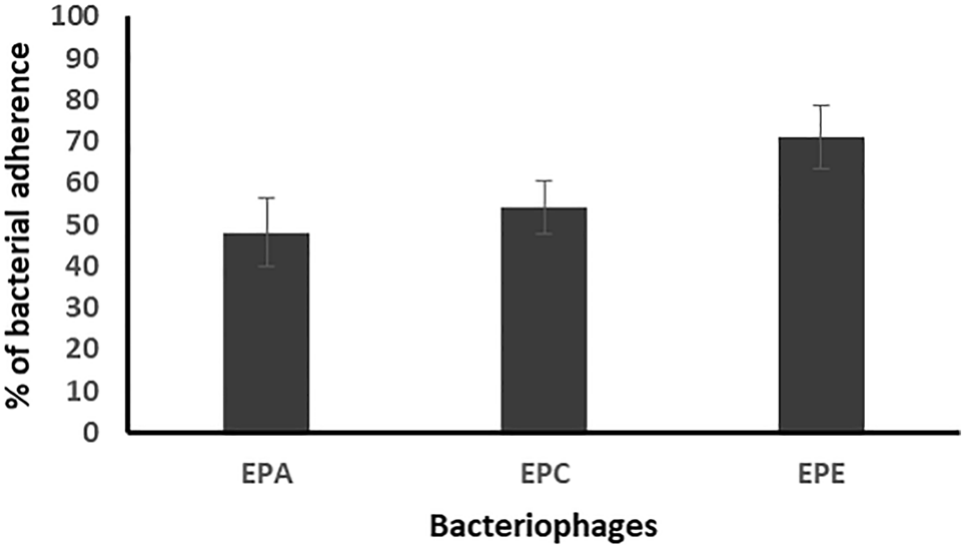
Percentage of bacterial adherence on the surface of catheters segments of the 3 enterococcal isolates (EF104, EF134, and EF151), prepared in dilution 1/500 from bacterial suspension adjusted to 0.5 MacFarland, after treatment the preformed biofilms with the three bacteriophages (EPA, EPC, EPE) as compared to the control. Each bar represents the mean of three independent measurements.

In addition, treatment of the enterococcal preformed biofilm on urinary catheter surfaces with the selected bacteriophages caused reduction in the number of adherent cells to 48.2, 54.2, 71.1%, respectively, as compared to that of the control (Fig. 8).

## Discussion

Urinary tract infections (UTIs) are one of the most frequent human bacterial infections. Enterococcal UTIs are of particularly concerned as they are radically resistant to first-line antimicrobial agents, especially vancomycin^16^. Also, *Enterococcus* strains have an extraordinary potential to form biofilms that are difficult to eradicate^17^. This fact together with the concern that humankind is reentering the “pre-antibiotics” era, have caused the development of alternative anti-infective practices to be one of the highest considerations of modern medicine.

Being one of the promising therapeutic approaches, phage therapy has several advantages that have made it potentially attractive over antibiotics due to phages’ high specificity, low toxicity to humans, self-amplification and liability to rapid modification to fight newly arising bacterial threats^18,19^. The specificity of phage action on host cell depends on the structure of the receptor on the bacterial surface that involved in phage adsorption which initiates the phage infection to the host bacterial cell and followed by process of phage replication and host cell lysis^20^.

In the present study, out of one hundred enterococcal urinary tract clinical isolates, 35% (n = 35) were found to form strong biofilm which increases the importance of finding a treatment for them. This result is close to that reported by Zhi-jian Yu et al*.* who isolated 113 samples from UTI patients and found 26.5% of them were strong biofilm forming^21^.

On screening for a specific enterococcal phage therapy, six bacteriophages were isolated from sewage water. Based on previous studies^22^, bacteriophages were successfully isolated from samples collected at governorate’s sewage site in Cairo, Egypt. This was attributed to the sewage rich contents of bacteria and organic matters^23^. On the other hand, no bacteriophage could be isolated from hospitals and clinics’ wastewater samples. This may be due to the excessive use of disinfectants in the hospitals and clinics that finally released into the wastewater, especially during the period of corona virus pandemic.

The increase in enteroccocal phage titers in the bacterial culture confirms the phage infection and replication in host cells, ending with bacterial cell lysis that is confirmed by plaque formation. The size of the formed plaques was varied (< 1–0.1–4 mm in diameter) after 24 h of incubation at 37 °C. In addition, hallow zones around the plaques were produced by only four bacteriophages (EPA, EPB, EPE, EPF) and the other two (EPC and EPD) showed plaques with defined boundaries. The isolated enterococcal bacteriophages showed lytic activity against seventeen *E. faecalis* isolates*,* five of which were sensitive to all the tested bacteriophages but the remaining isolates showed different sensitivity patterns. The lytic effect of tested bacteriophages on biofilm associated *Enterococcus* isolates gave an indication for their ability to penetrate the biofilm reaching to the host bacterial cells, in agreement with the previous reports that showing the efficacy of bacteriophage to reduce bacterial biofilm^24^.

Upon characterization using TEM, four of the isolated phages (EPA, EPB, EPD, EPF) having long and contractile tails, were found to be related to the family *Myoviridae* and the other two bacteriophages (EPC and EPE), having long, flexible and non-contractile tails, are related to family *Siphoviridae*. This finding gave another support that such phages are effective against the *Enterococcus* species, since most of the viruses related to both *Myovirdae* and *Siphoviridae* families are potential predators of enterobacteria, according to the international committee on taxonomy of viruses^25^. In addition previous studies identified different bacteriophages related to family *Myoviridae*^26,27^ and *Siphoviridae*^22,28^ showed promising antibiofilm activity against different types of biofilm associated bacteria.

Regarding the thermal and pH stability, all the tested *E. faecalis* phages showed excellent stability at temperature between 40 and 70 °C for 1 h. Whereas, three bacteriophages (EPD, EPE, EPF) survived at 90 °C and only one bacteriophage (EPD) retained its lytic activity at 100 °C. Moreover, all the bacteriophages showed stability at pH between 3 and 11. The stability of the tested phages at wide range of temperature and pH gave them advantage for preparation in suitable pharmaceutical form and used for therapeutic purpose. In addition, the stability of the phage at acidic and alkaline conditions (pH 3–11) facilitates the product to be administered through oral route without any change in the viability along the gastrointestinal tract.

Our findings nearly conceded those reported by a previous work that evaluated stability of *Staphylococcus* bacteriophages at different temperature and pH values^29^. However, *E. faecalis* phage PHB08 isolated by Yang et al. showed stability at temperature and pH ranges of 4–60 °C and 5.0–9.0, respectively^30^. Also, ValSw3-3 bacteriophage displayed a stability at temperature between 4 and 50 °C and pH between 3 and 10, respectively^31^. In addition, Goodarzi et al*.* investigated that the isolated enterococcal phage S2 showed stability when exposed to a wide range of pH values (1 to 11)^22^.

For studying the antibiofilm activity of the enterococcal phages, four different bacterial dilutions (1/1000, 1/500, 1/100, 1/10) were used. The results revealed that all the tested phages can prevent the biofilm formation and eradicate the preformed ones of the respective *Enterococcus* isolates. The averages of the formed biofilms were 49.5%, 41.4%, 52.6%, and 49.3% for bacterial dilutions 1/1000, 1/500, 1/100, and 1/10, respectively, as compared to that of the control. Also, treatment of preformed biofilm by the same dilutions caused reduction in the biofilm to 88.1, 75.6, 82.3, 85.1%, respectively, as compared to the control. Accordingly, the best antibiofilm activities induced by tested bacteriophages were obtained on using bacterial dilution 1/500 at which the percentage of the remained biofilm was significantly lower than that of all the other dilutions (*p*-value < 0.05). In addition, the three enterococcal bacteriophages (EPA, EPC and EPE) had the strongest antibiofilm activity. Therefore, bacterial dilution 1/500 was used for evaluating the efficacy of the three bacteriophages EPA, EPC and EPE to prevent adherence of three of strong biofilm producer enterococcal isolates (EF104, EF134, and EF151) to the surface of urinary catheter segments. The obtained results confirmed the ability of the selected bacteriophages to prevent biofilm formation and adherence of the tested isolates onto the surface of the urinary catheter through reducing the number of the adherent cells to an average of 30.8, and 35.3, 43.8%, respectively, as compared to that of the control. In addition, they efficiently eradicated the pre-adherent cells on the catheter surface and reduced their number to the average of 48.2, 54.2, 71.13%, respectively, as compared to the control.

The ability of the tested enterococcal tailed phages to invade the biofilm matrix and cause lysis to the bacterial cells in the deeper layer results from the hydrolytic activity of depolymerase enzymes that present in their tail spike proteins^32,33^ or the presence of endolysins that have remarkable activity to lyse planktonic cells^34,35^. Accordingly, the findings of this study support the notion that bacteriophages can be used as a promising alternative strategy to conventional antibiotic therapy to prevent and treat the UTIs and also urinary catheter associated infections caused by biofilm producing *E. faecalis* pathogens.

## Materials and methods

### Collection of *Enterococcus* isolates and culture conditions

One hundred enterococcal clinical isolates were obtained from different Microbiology Laboratories in Cairo (Al-Borg laboratories) and Alexandria (Al Shatby Hospital). The obtained isolates were taken after the laboratories finished the analysis process on these isolates. According to the obtained information, all the isolates were *Enterococcus* species and isolated from urine samples of urinary tract infected patients. Purification and identification of the collected isolates were done by surface streaking on Bile Esculin agar (oxoid), microscopical examination, and catalase test^36^. VITEK system (Biomerieux, USA), was carried out at El Zahraa University teaching hospital to identify the species of some of the obtained enterococcal isolates.

The isolates were stored at −80 °C in 25% glycerol in nutrient broth. Before use, the isolates were plated from glycerol stocks onto Luria Bertani (LB) agar and incubated at 37 °C for 24 h.

### Assessment of biofilm formation by microtiter plate assay

For preparation of fresh bacterial suspension, one colony from the isolate grown on LB agar plates was inoculated in 5 mL of tryptone soy broth with 1% glucose (TSBG) and incubated at 37 °C for 24 h. The obtained cultures were first adjusted to 0.5 MacFarland (1 × 10^8^ CFU/mL) using spectrophotometer V-630 (Unicam, UK) and then diluted 1:100 with fresh medium (10^6^ CFU/mL). Individual wells of sterile 96 wells flat bottom tissue culture plate were filled with 200 µL of the diluted cultures and incubated at 37 °C for 24 h. After incubation, contents of each well were removed by gentle tapping, then washed with 0.2 mL of phosphate buffer saline (PBS, pH 7.2) three times to remove planktonic cells. Biofilms formed by adherent bacteria were fixed by methanol and stained by 0.1% crystal violet solution (Oxford, UK). Excess stain was removed by using deionized water and plates were kept for drying. The dyes adhered to bacterial cells were resolubilized by 33% glacial acetic acid (Fisher Chemical, US). Optical density (OD) of stained adherent biofilm was obtained by using micro-ELISA auto-reader (ELx-800, BiotTek, UK) at wavelength 630 nm. Wells containing bacteria free medium were used as controls^37^. Measurements were performed in triplicate and the average was calculated. As described by Mathur et al., the strength of the biofilm formation was classified into strong (OD > 0.240), moderate (OD = 0.120–0.240), and weak and non-biofilm producer (OD < 0.120)^38^.

### Isolation and purification of bacteriophages from sewage samples

Twelve sewage samples were collected from different locations in Egypt; hospitals (Al Shatby, Alexandria and El Kasr El ainy, Cairo, Egypt), clinics and community (Kalyoub and Zagazig cities, Egypt). Sewage samples (4.5 mL) were mixed with 0.5 mL of overnight culture of *Enterococcus sp.* in tryptone soy broth (TSB) and 5 mL fresh double strength TSB in falcon tube and incubated in incubator shaker at 37 °C and 150 rpm. Enriched samples were then centrifuged (Sigma 2-16 KL refrigerated centrifuge, Germany) at 6700×*g* for 20 min at 4 °C. Filtration of the supernatant was done using a 0.45 μm pore-size disposable syringe filter (Cellulose acetate, sterile) to remove any remaining bacterial cells. Screening for presence of phages by plaques demonstration was done using the double layer agar (DLA) method as described by Frederick^39^. In brief, 100 µL of the bacteriophage and 200 µL of overnight bacterial samples were added to 3 mL of sterile tryptone soy soft agar (TSA) and mix gently then poured on 10 mL hard TSA in petri dish. After solidification, the plates were incubated in inverted position at 37 °C for 24 h to visualize the developed plaques.

To obtain a purified bacteriophage, sterile tip was gently stabbed in the focal point of a single plaque and then put into 1 mL of TSB and pipetted to deliver bacteriophage particles into broth. The filtrate was ten times diluted and used to proceed in DLA method to observe the formed plaques. The same steps of isolation were repeated for purification and enrichment. This method was rehashed until pure plaques appeared^40^.

### Characterization of bacteriophages

#### Determination of host range of infection

An aliquot (10 µL) of phage sample was spot inoculated onto Tryptone soy DLA seeded with one of the strong biofilm producers *enterococcal* isolates. The plates were then incubated inverted for 24 h at 37 °C and the plaques formation examined on the next day^41^.

#### Plaque morphology

Plaques morphology was characterized through determination of diameter size, clearance and presence of halo zone around the plaques^42^.

### Effect of temperature on bacteriophages’ stability

Thermal stability of bacteriophages was measured by exposure of the enterococcal phages to five different temperatures: 40, 50, 70, 90 and 100 °C for 1 h. Then spot test method was carried out for checking the survival of bacteriophages^29^.

### Effect of pH on bacteriophages’ stability

Enterococcal bacteriophages were exposed to different pH values ranging from 3 to 11 using 0.1 M NaCl and 0.1 M HCL, at temperature of 37 °C for 1 h. Then, spot test method was carried out for checking the survival of bacteriophages^29^.

### Transmission electron microscope (TEM) visualization

An aliquot (5 µL) of phage lysate (10^10^ PFU/mL) was transferred to carbon grid and air dried for 5 min, then 5 µL of negative stain phosphotungstic acid was added and air dried for 10 min. Images of the phages were captured, at Cairo university research park, using transmission electron microscope (JEM-1400 EM, Japanese Electron Optic Ltd.) which was set at 60 kV and magnification power of 10,000×^42^.

### Antibiofilm effect of bacteriophages

#### Prevention of biofilm formation

Fresh culture of strong biofilm producer *E. faecalis* isolate was used to prepare bacterial suspension in TSBG broth and adjusted to 0.5 MacFarland as described before and then diluted to 1/10, 1/100, 1/500 and 1/1000 with fresh medium. Individual wells of sterile 96 well flat bottom tissue culture plates were filled with 100 µL of the diluted cultures and 50 µL of bacteriophages of titer 10^7^, then the plates were incubated at 37 °C for 24 h. After incubation, the wells’ liquid contents were aspirated and the process of washing, staining and measuring the OD of the adherent biofilm was carried out as described above. Wells contained TSB inoculated with tested bacterial isolate were used as control^24^.

### Treatment of preformed biofilm

As described before, 200 µL of diluted bacterial culture in TSBG (10^5^ CFU/mL) was used to fill each well in 96 well flat bottom plates and incubated at 37 °C for 24 h. After incubation, the liquid media was aspirated and the wells were washed thrice using PBS to remove any planktonic cells, followed by adding 200 µL bacteriophage lysate with titer 10^7^ and incubated for 24 h. The phage lysate was removed after incubation and the wells were washed, stained, and OD of the remaining biofilm was determined. Wells contained only TSB inoculated only with tested bacterial isolate were used as control^24^.

### Effect of bacteriophage on bacterial adherence to urinary catheter surfaces

#### Prevention of bacterial adherence to catheter surfaces

Three pieces (each 1 cm length) of urinary catheter segments (size 14) were placed in Wassermann tubes containing 0.5 mL of phage lysate with titer 10^7^ for 1 h at 37 °C, then 1 mL of bacterial suspension adjusted to 0.5 McFarland and diluted 1:500 with TSBG was added and continue incubation for 24 h. After incubation, each catheter segment was removed and washed thrice with PBS to remove planktonic cells and then placed in new Wassermann tube with 1 mL saline. For dislodgment of the adherent cell from catheter surface, the segment in saline was sonicated for 30 s three times at 20 kHz, then counting of the released cells was carried out by drop plate method. For each sample, counting of the viable bacterial cells was carried out in triplicates and the mean value was calculated. The segments that were placed in bacterial suspension without previous treatment with phage lysate were used as control^43,44^.

#### Eradication of preformed bacterial biofilm on catheter surfaces

Three pieces of urinary catheter segments were incubated with 1 mL bacterial suspension for 24 h at 37 °C to form bacterial biofilm on catheter surfaces. After incubation, the segments were removed and washed with PBS three times to remove planktonic cells and each segment was transferred to new tube containing 1 mL TSBG inoculated with phage lysate with titer 10^7^ and incubated for 24 h at 37 °C. After incubation, the number of adherent bacterial cells to segment surface was determined as described before. The segments that were placed in bacterial suspension without treatment with phage lysate were used as control^45,46^.

### Statistical analysis

The obtained data were converted to percentage as compared to that of the control. Then statistical analysis (one-way ANOVA test) was carried out by using GraphPad Prism (GraphPad Soſtware Tools, Inc., La Jolla, CA, USA) to evaluate the effect of the isolated bacteriophages on the biofilm associated *E. faecalis* urinary tract pathogens. Data were presented as mean ± standard deviation and a p-value less than 0.05 was considered statistically significant.

### Ethical approval

This article does not contain any studies with human participants or animals performed by any of the authors.

## Supplementary Information

Supplementary Tables.

## Data Availability

The datasets generated during and/or analyzed during the current study are available from the corresponding author on reasonable request.
