# Supplementary material for: Characterization and anti-biofilm activity of bacteriophages against urinary tract Enterococcus faecalis isolates
Source: Sci Rep. 2022 Jul 29;12:13048. doi: 10.1038/s41598-022-17275-z (PMC9336127; doi:10.1038/s41598-022-17275-z)
Supplement: Supplementary file 1 — Supplementary Tables. [file 41598_2022_17275_MOESM1_ESM.docx]

**Supplementary information
Characterization and Anti-biofilm Activity of Bacteriophages**

**Against Urinary Tract *Enterococcus faecalis* Isolates**

Doaa M. El-Atrees^1^, Reham F. El-Kased^1^, Ahmad M. Abbas^2,3^, Mahmoud A. Yassien^2*^

^1^ Department of Microbiology, Faculty of Pharmacy, The British University in Egypt (BUE), El-Sherouk City, 11837, Cairo, Egypt. 0000-0001-6964-2560

^2^Department of Microbiology and Immunology Faculty of Pharmacy, Ain Shams University, African Union Organization Street, Abbasia, Cairo11566, Egypt.

^3^ Department of Microbiology and Immunology, Faculty of Pharmacy, King Salman International University, Sinai, Egypt.

* Prof. Dr. Mahmoud A. Yassien,

Microbiology and Immunology Department, Faculty of pharmacy, Ain Shams University, African Union Organization Street, Abbasia, Cairo11566, Egypt.

<Tel:+202-01001503920>

ORCID #: <https://orcid.org/0000-0002-0310-8098>

Email: myassien61@yahoo.com

**Table S1**: Optical density averages of the strong biofilm forming

*E. faecalis* isolates with **±** standard deviation.

| **Isolate codes*** | **Average OD**  **value ± SD** | **Isolate codes** | **Average OD value ± SD** | **Isolate codes** | **Average OD value ± SD** |
| --- | --- | --- | --- | --- | --- |
| EF11 | 1.17±0.05 | EF50 | 0.39±0.03 | EF115 | 0.33±0.1 |
| EF14 | 1.18±0.08 | EF54 | 0.41±0.02 | EF116 | 1.24±0.3 |
| EF16 | 0.26±0.03 | EF55 | 0.263±0.03 | EF124 | 0.44±0.04 |
| EF19 | 1.18±0.1 | EF80 | 0.28±0.05 | EF125 | 0.244±0.02 |
| EF20 | 0.64±0.08 | EF99 | 0.46±0.07 | EF133 | 1.346±0.1 |
| EF33 | 0.45±0.03 | EF101 | 2.3±0.1 | EF134 | 2.34±0.1 |
| EF37 | 0.29±0.02 | EF103 | 1.05±0.5 | EF141 | 0.53±0.05 |
| EF38 | 1.28±0.37 | EF104 | 2.57±0.2 | EF143 | 1.91±0.12 |
| EF43 | 0.89±0.02 | EF105 | 0.36±0.2 | EF146 | 0.43±0.01 |
| EF44 | 0.38±0.04 | EF112 | 0.71±0.09 | EF149 | 0.61±0.06 |
| EF46 | 1.41±0.013 | EF113 | 0.24±0.01 | EF151 | 2.35±0.26 |
| EF48 | 0.27±0.02 | EF114 | 1.27±0.06 |  |  |

Isolate codes (EF)* are the codes for *E. faecalis* isolates.

**Table S2**: Effect of different temperatures (40-100 °C) on the lytic activity of the 6 enterococcal phages (EPA: F) against the 5 enterococal isolate that are sensitive to all the tested bacteriophages; EF 38, EF 43, EF 101, EF 133 and EF 134.

| Bacteriophages | | | | | | |
| --- | --- | --- | --- | --- | --- | --- |
| Temperature (°C) | EPA | EPB | EPC | EPD | EPE | EPF |
| 40 | + | + | + | + | + | + |
| 50 | + | + | + | + | + | + |
| 70 | + | + | + | + | + | + |
| 90 | - | - | - | + | + | + |
| 100 | - | - | - | + | - | - |

(+): presence of bacteriophages plaques (clear spot), (-): absence of bacteriophages plaques (no lysis).
